# Outcome of a Multi-modal CBT-based Treatment Program for Chronic School Refusal

**DOI:** 10.1177/2333794X211002952

**Published:** 2021-03-30

**Authors:** Johan Strömbeck, Robert Palmér, Ia Sundberg Lax, Jonas Fäldt, Martin Karlberg, Martin Bergström

**Affiliations:** 1 Åbo Akademi University, Turku, Finland; 2 Magelungen Utveckling AB, Stockholm, Sweden; 3 Uppsala University, Uppsala, Sweden; 4 Lund University, Lund, Sweden

**Keywords:** school refusal, treatment outcome, CBT, anxiety, depression

## Abstract

School refusal (SR) can have several negative consequences, but effective treatments are available. When chronic, school absence requires comprehensive treatment. This study evaluates an intervention for SR based on a Cognitive Behavioral Therapy (CBT) model, *Hemmasittarprogrammet* (HSP). Attendance, anxiety, depression, quality of life, and emotional and behavioral symptoms were measured at pre-treatment, post-treatment, and follow-up. The participants (n = 84; 69% male) were SR students between 10 and 17 years old and their parents. School attendance increased after treatment and at follow-up. The proportion of students totally absent from school decreased and the number of students with an acceptable level of school attendance increased. Levels of anxiety and depression were lower both post-treatment and at follow-up for the youths and their parents. HSP, a promising treatment program for school refusal, builds on the literature of CBT-based programs, which has been shown to be effective for SR treatment. However, more research about the effectiveness of the program is needed. Future studies should have a stronger research design, include a measure of fidelity, and be evaluated independent of the founders of the program under investigation.

School refusal (SR) means students are reluctant to attend school and occasionally refuse to attend school. Often, these students find it difficult to leave the safe environment of their homes as even the mere thought of going to school is a source of stress. SR students show no tendencies for antisocial behavior, except when they are coerced to go to school. Usually, SR students do not try to hide their absence from their parents. In addition, the parents of these students have made attempts to encourage their children to attend school.^1^

Studies from outside of Sweden show a prevalence of SR of around 1% to 2%.^2^ Although school attendance in Sweden is compulsory and regulated by the Education Act,^3^ not all youth actually attend school. In Sweden, tardiness and occasional absenteeism is a problem. For some students, both unexcused (ie, absences without an excuse from parents) and excused absences can become a problem.^4^ Studies in Sweden show that almost 1700 students have unexcused continuous absences over a month or more and over 18 000 students have unexcused repeated occasional absences over 1 month.^5^ Some studies, however, have reported even higher absence numbers.^6^

Unexcused continuous absence is as common among boys as girls and is roughly as common in private/independent as municipality schools. According to the Swedish National Agency of Education,^7^ absenteeism tends to be higher in grades 7 to 9 (1345) than in grades 1 to 6 (302), a pattern that is also evident in the intervention program investigated in this study, *Hemmasittarprogrammet* (HSP).

School attendance is essential for student success as well as for social and personal development.^8^ Children who are absent from school are at risk for negative outcomes such as mental health problems, somatic health problems, and addiction.^2,7,9-12^ Therefore, school attendance is fundamental to success later in life.^13^

Without treatment, SR places students at risk for chronic school absenteeism; however, help is available. A recently published systematic review reveals that interventions for SR can increase school attendance.^14^ When school absence persists, secondary problems such as anxiety and fear become not only a result of absenteeism but also a cause of absenteeism.^4^ Therefore, SR interventions such as HSP need to be intensive, multimodal, and include the family, educators, and other professionals.^15^ This study evaluates the effectiveness of HSP, a CBT-based psychosocial intervention for students with prolonged school absenteeism. HSP’s primary goal is to help SR students return to school long-term. The focus of this study is HSP’s outcomes: increased school attendance and improved mental health.

According to the Swedish School Inspection,^16^ several factors influence the effectiveness of whether SR students return to school: early identification, assessment, commission, measures, and cooperation. These factors have been emphasized by *Skolverket*^7^ and Heyne and Rollings.^17^ The importance of a complex intervention also becomes more significant when absenteeism is prolonged such as over an entire semester.^15^ In a meta-analysis, Maynard et al^18^ compiled the effects of psychosocial treatment interventions of 8 interventions (7 were CBT based). The interventions improved school attendance, but anxiety levels were as high after as before treatment. Another CBT-based treatment is HSP. This study examines whether any change in school attendance and mental health is evident immediately after the intervention and at a 6 month follow-up.

Long and extensive absenteeism is a significant problem in today’s school. High absence rates affect not only the youths but also their families and the network around the families. This can lead to an increased risk of negative consequences in life, such as lack of grades, mental health problems, and economic deprivation. It is known that absenteeism is related to school factors, individual factors, and social factors. To deal with the absence, adjustments can be done in school. For example, a rigorous school attendance record system seems to improve attendance; however, when absence rates increase, the interventions need to be more extensive. Some evidence exists for the effectiveness of short-term CBT for SR.^14,18^ Previous studies show these programs improve school attendance, but do not affect anxiety, so more follow-up studies are needed to see whether anxiety decreases over time.^14^

HSP, which started in 2012, has not been systematically evaluated for its effectiveness. The only evaluation so far shows a positive change after treatment. This study addresses caregivers’ desire to control and develop the quality of their treatment center, which should include evaluation or systematic follow-up.^19^ Furthermore, more knowledge is needed about the effects of interventions used by social workers.^20,21^ In this study, the outcome of a Swedish CBT-based intervention directed toward excessive and prolonged school absenteeism is evaluated by measuring school attendance and the mental health of the youths and their parents at pre-treatment, post-treatment, and the 6-month follow-up.

This study evaluates the effects of HSP. Primary outcome measure was attendance. Secondary outcome measures were emotional and behavioral symptoms for the children and their parents. We hypothesized that school attendance would increase, and emotional and behavioral symptoms would decrease for the children as well as their parents.

## Method

### Participants

The treatment was managed by Magelungen Development Inc. (MDI). MDI is an employee-owned company that was founded 1993 and consists of manual-based treatments for children and adolescents, residential care, and day treatment centers as well as alternative educational programs and resource schools. The participants were referred to HSP for SR by the local social services. Referrals, which came from all over Sweden, placed students in 1 of 11 MDI treatment units. At the beginning of the intervention, families were asked to participate in the study; that is, participants were recruited to the study through MDI. The inclusion criterium was treatment length longer than 4 months.

The study included 84 youths referred to HSP between 2012 and 2018. The youth participants (69% male) were 10 to 17 years old (*M* = 14.1), and their parents (1 foster parent and 1 other relative). 74 youths, 79 mothers, and 60 fathers participated at pre-treatment; 59 youths, 55 mothers, and 35 fathers participated at post-treatment; and 39 youths, 37 mothers, and 26 fathers participated at follow-up.

A majority (76%) did not attend school at all before treatment and their school attendance had been low for a long time: 54% had been absent more than 50% of school during the previous 2 years and 27% had been absent more than 50% for 1 to 2 years. Those who went to school had a modified schedule; only 24% had more than 15 lessons per week (full time school schedule in Sweden counts as 25 lessons per week for youths aged 13-16). Before treatment, the attendance rate was generally low; only 4% went to school more than 30% of the time.

Information about psychiatric or neuropsychiatric diagnoses were noted in 25 of the initial cases. Of these 25 adolescents, 20 had 1 diagnosis or more: 12 with autism spectrum disorder, 3 with ADD, 1 with ADHD, 13 with depression or anxiety disorder, 2 with selective mutism, and 1 with oppositional defiant disorder.

Treatment length varied between 4.7 and 20.5 months (*M* = 11.9). After treatment, the youths were enrolled in resource schools, a combination of treatment and regular school, or regular school with a modified schedule. The intervention ended after the first phase of treatment for approximately 10% of the intention-to-treat sample.

### Procedure

The study was a 1 group pre-test/post-test design^22^ with an additional measurement made at a 6-month follow-up. Measures were collected at pre-treatment (ie, at the beginning of the treatment; T1), post-treatment (T2), and at a 6-month follow-up (T3). Youth and parent both answered the questionnaires at all 3 measuring points (T1-T3). Those who did not participate at T1 did not participate at T2 or T3. Those who participated at T1 but not at T2 still participated at T3. The present study is part of the routine follow-up managed by MDI for all internal treatment programs. During one of the first meetings, families were asked to participate after being informed of MDI’s follow-up protocol.

During the initial assessment, the participants were given written information about the study. If they agreed to participate, they were given a consent to participate form and questionnaires. Parents could either complete the form and questionnaires at the treatment center or at home. Treatment staff were available to assist if anything concerning the questionnaires or the evaluation procedure was unclear. At the start of treatment, the family was informed of the follow-up procedure, including information about the 3 measuring points. The participants were encouraged to complete the pre-test and post-test at the treatment unit, but the follow-up had to be administered in a different way: treatment staff had to contact the family through e-mail, phone, or post to set up a meeting. This meeting could take place at home or in another place (eg, a café). The questionnaires could also be mailed home to be answered and returned by the child and the parents.

The participants provided written informed consent. They were also informed of the principles of research ethics^23^ and that their participation was voluntary. They were also assured that whether or not they participated in the evaluation would not affect the quality or content of the intervention.

### Ethics Approval and Informed Consent

Ethical approval for this project was given by the Swedish Ethical Review Authority (2019–00078).

### Measures

The primary outcome measure was school attendance as this was the primary aim of the intervention. Secondary outcome measures were emotional and behavioral symptoms and quality of life for both the youths and their parents. That is, both children and parents often suffer from mental health problems due to SR.^18^ In addition, outcomes of interventions targeting SR is usually assessed by measuring school attendance and anxiety.^18,24^

Attendance was measured in 2 ways and recorded in a prepared format: in an earlier version of the form (Version 1), the number of lessons during the previous 3 weeks and the number of lessons that the student attended each school day were recorded. In a later version (Version 2), attendance was recorded by asking for the number of lessons the student had scheduled every week and what percentage of these lessons the student attended during the previous 6 months. This information was gathered primarily from the school’s documentation system, but if this information was not available, then a parent or a teacher were asked to estimate the percentage of these lessons the student had attended. When summarizing these data, attendance rates were converted into a percentage of full-time school (ie, 25 lessons per week).

The Strengths and Difficulties Questionnaire (SDQ) is a short behavioral screening instrument that consists of 25 items divided into 5 subscales: emotional symptoms, conduct problems, hyperactivity/inattention, peer relationship problems, and prosocial behavior. Prosocial behavior, an umbrella term, includes several interpersonal behaviors about care for others (range 0-10). The other 4 subscales are summed to create a total difficulty score of 20 items (scale 0-40).^25,26^ An extended version of the SDQ also includes an impact supplement with questions about chronicity, distress, social impairment, and burden to others. Five of these items are used to create the variable Impact score (0-10) or Impact Factor score (0-15). The self-report version and parent-report version was used. Internal consistency (Cronbach’s α) varied at T1 from unacceptably low^22^ to very good 0.45 (Peer relationship problems in the self-report version) and 0.85 (Prosocial behavior, self-report). However, most of the subscales were adequate or almost adequate (around 0.60-0.75). Inter-rater reliability between parent and youth varied between 0.32 and 0.50 (emotional symptoms = 0.32; Conduct problems = 0.41; Hyperactivity = 0.48; Peer relationship problems = 0.50; Prosocial behavior = 0.49; Total score = 0.48).

The Hospital Anxiety and Depression Scale (HADS)^27^ was used to assess anxiety and depression. HADS contains 7 items about anxiety and 7 items about depression, both rated on a 4-point scale (total anxiety/total depression score; 0-21). Two cut-off scores exist: possible cases (7) and probable cases (11). Internal consistency levels were adequate to very good (α = 0.78-0.87).

Youth and parents reported their quality of life using the Ladder of Life, a short rating scale encompassing 3 questions regarding current, past (1 year ago), and future (1 year from now) life satisfaction, ranging from 1 (worst possible life) to 10 (best possible life).^28,29^

### Treatment

*Hemmasittarprogrammet* (HSP), a multimodal and manual-based treatment program, is designed to increase school attendance and decrease anxiety, depression, and other psychiatric symptoms.^30,31^ The target group is elementary and secondary school students with severe, chronic, and complex school absenteeism.

HSP incorporates treatment elements at different levels: the individual, parent, and school. HSP includes individual components for the youth (eg, skills training, social skills training, gradual school approach, behavioral activation, and problem solving) and for the family (eg, regular meetings with the parents that focus on rules, agreements, daily routines, psychoeducation, and conflict reduction strategies). Treatment staff also meet with teachers and other school staff to explain the student’s specific needs and problems and help them adapt the pedagogic and social environment to address these needs.

HSP is a program created to address long and extensive absence from school. Most children and teenagers attending the program have been totally absent for at least 6 months, and the problem with school attendance has lasted for 1 to 2 years. There are more boys than girls, most are 13 to 15 years old, and ADHD and autism spectrum disorders are common.

The clinical experience is that the target group could be included in the definition of SR.^32^ The intervention is about 12 months long and is divided into 3 phases: (1) assessment phase (3-4 weeks); (2) treatment phase (6-9 months); and (3) maintenance phase (around 3 months). During the assessment phase, the treatment team meets with the child, the parents, and school staff to gather information about the school absence, the family system, and the child’s strengths and difficulties. In addition, relevant documentation is assembled (eg, assessments or initial evaluations from a psychiatric clinic or the social services). Information is also gathered through interviews, behavior analysis, and standardized assessment methods. The flexibility of the program allows the treatment to be individualized. This phase is important for building a working alliance with the family and the school.

During the treatment phase, treatment goals are finalized in writing, with the primary goal to increase school attendance gradually. This phase is guided by the behavior analysis and the goals from the assessment phase. The treatment phase focuses on behavioral change in those situations where the problematic behaviors occur, often in the child’s home or school environment. There are several standard elements of the treatment: psychoeducation (eg, about anxiety); information about what is going to happen and why (rational), that is, gradual re-introduction into school (eg, meeting with teachers after school hours and going to the school by car but staying in the parking lot); involvement of parents (eg, by helping them acknowledge and reinforce progress of the child); and plans for the return to school with school staff (eg, teachers, principals, and the student health team). Additional treatments could be added based on the individualized joint treatment document created during the assessment phase. These treatments could involve social skills training, different exposure techniques, and behavioral activation. Treatment is also adjusted when mental health problems are present (eg, anxiety and depression). When the family is better equipped to handle situations and practice their skills, the third phase begins—the maintenance phase.

The maintenance phase intends to create a lasting effect. During this phase, the treatment team supports the family more via telephone and e-mail. As the team’s interaction with the children and their parents decreases, the child, the family, and the school have more opportunities to practice the skills they learned on their own, a crucial step in ensuring lasting results. In addition, an important part of the intervention is coordination between different service systems (eg, child psychiatry and social services). A good alliance with the child and parents is also emphasized as well as a thorough assessment. Initially, the support for the family is intense, but the program is flexible to meet the individual needs of the target group.

According to the theory of change of the program, a positive outcome—where the youth reach an acceptable level of school attendance, the youth and parents have improved mental health, and the family climate improves—should be obtained if the relationship between the therapist and the family is good, if the youth and the parent have gained knowledge about their situation and have agreed on the content and the goal of treatment (ie, a good working alliance), if the coordination between home, school, and treatment team has worked, if the family has learned new strategies, if the youth has gained new social skills, and if adjustments have been made in school.

### Statistical Analyses

Initially, independent sample *t*-test was used to assess whether the group that only participated at T1 were different from those who participated at T1 and T2 and/or T3. Attendance rates were reported as a change in mean for the group as well as the percentage of the sample with an acceptable level of attendance. An acceptable level of school attendance has previously been operationalized in different ways—for example, >80% attendance^33^ or >90% attendance.^34^ Paired sample *t*-tests were used to estimate changes over time, and effect size was obtained using Cohen’s *d*: < 0.20 = no effect; 0.20 to 0.49 = small effect; 0.50 to 0.79 = medium effect; and >0.80 = large effect.

Emotional and behavioral symptoms were reported both at the group level and by counting the number of participants over the cut-off. The cut-off for SDQ was estimated using the 90th percentile.^35,36^ For HADS, the initial 2 cut-offs from Zigmond and Snaith^27^ were used: >7 = possible anxiety or depression and >11 = probable anxiety or depression. No imputation method was used as selection was based on pairwise deletion.

## Results

### Missing Data

**Figure 1.**
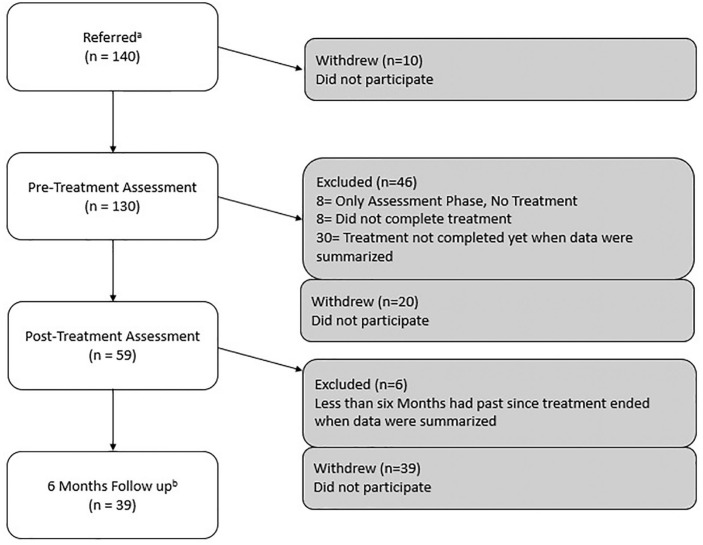
Participant flow. ^a^Estimated from data of attrition during 2015 to 2018 when 93% of youths in treatment participated. ^b^Of those who participated at T3, 34 had also participated at T2 and 5 had not.

External attrition at pre-test (T1) was 7%, at post-test (T2) 37%, and at follow-up (T3) 56%. Between 2012 and 2018 (ie, the study period), 93% of the youths and/or their parents participated at pre-test, which is approximately 130 of 140 cases (because a lack of administration system the number of cases and attrition rate was not documented before year 2015 and thereby had to be estimated) (Figure 1). Among those who participated at the pre-test (T1), 8 did not complete treatment so the intervention was changed to include only the assessment period, 8 completed the intervention for no longer than 6 months and therefore they were excluded from further assessments, and 30 were still in treatment when the data for the present study were being summarized and processed. Therefore, 84 cases were included in the final analysis. Among these cases, 59 youths participated at T2 and 39 at T3. Among those who participated at follow-up, 34 had participated at T2 and 5 had not participated at T2.

The results do not seem to be influenced by selection bias with respect to the difference between those who participated only at T1 compared to those who participated also at T2 and/or T3. Selection bias was tested by comparing the total SDQ score of the groups (youth), the Ladder of life—present time score (youth), and HADS (youth and parent). According to the independent sample *t*-tests, there is no reason to believe that the samples were not drawn from the same population (*P*-values varied between .08 and .98).

Missing items were not statistically significant; at T1, 74 youths answered the SDQ. At most, 4% of item 23 was missing: “I get on better with adults than with people my own age.” In addition, items 26 to 26d in the SDQ (the impact supplement) were missing for 4% of youths who answered this at T1. The number of missing items were otherwise lower than 4%.

### Attendance Rates

#### Version 1—Attendance in school previous 3 weeks

Attendance reported during the previous 3 weeks increased over time. Before treatment, attendance was 6.2% of full-time, after treatment 18.1%, and at follow-up 30.3% (Table 1). These numbers correspond to 1.6 lessons per week pre-treatment, 4.5 lessons per week post-treatment, and 7.6 lessons per week at follow-up.

**Table 1.** School Attendance during the Previous 3 weeks (Version 1) and the Previous 6 months (Version 2) Stated in *M* (SD), Interquartile Range, and Values of Minimum and Maximum.

	Pre (T1)	Post (T2)	Follow up (T3)
Version 1—attendance (%) of full time *M* (SD) during previous 6 months			
*M* (SD)	6.2 (11.8)	18.1 (19.9)	30.3 (27.5)
Q1-Q3	0.0-9.3	0.0-29.0	5.0-42.7
Min-max	0.0-48.0	0.0-81.3	0.0-100.0
Version 2—attendance (%) of full time *M* (SD) during previous 3 weeks			
*M* (SD) 2.5 (6.5)	16.8 (27.2)	26.5 (31.8)	
Q1-Q3	0.0-0.0	0.0-30.4	0.0-36.0
Min-max	0.0-32.0	0.0-100.0	0.0-94.0

#### Version 2—Attendance during the previous 6 months

Attendance during the previous 6 months increased over time. Before treatment, attendance was on average 2.5% of full-time (about 0.6 lessons per week), whereas after treatment and at follow-up attendance was 16.8% of full-time (about 4.2 lessons per week) and 26.5% (about 6.6 lessons per week), respectively.

Attendance rates did not increase for everyone: some students did not attend school after treatment. The proportions of participants who were totally absent from school were 76% before treatment, 41% after treatment, and 27% at follow-up. For several of the participants, the schedule was individualized, with a reduced number of lessons at post-treatment and follow-up. Many participants also had a reduced schedule at T2 and T3; that is, only 13% at T2 and 30% at T3 had more than 15 lessons per week.

Cut-offs for what counts as normal or acceptable levels of school attendance have been operationalized differently; for example, Blagg and Yule^33^ defined acceptable levels as 80% and Kearney and Silverman^34^ defined acceptable levels as 90%. In the present study, 5% reached the 80% cut-off after treatment and 19% at follow-up. School attendance of 90% or more was reached by 5% at post-treatment and 7% at follow-up. Before treatment (pre-treatment assessment), no one (0%) reached acceptable levels of school attendance.

### Self-reports of Youth Mental Health

Youth mental health improved over time, where all comparisons except 1 (quality of life 1 year ago) indicated better mental health—decreased symptom burden, lower anxiety and depression, and higher quality of life (Table 2). Effect sizes varied between 0.03 and 0.63. Of the 24 comparisons, 6 were counted as non-existing or having no effect (under 0.20), 15 as small (0.20-0.49), 3 as medium (0.50-0.79), and none as a large effect (over 0.80).

**Table 2.** Self-Report Youth.

		T1 (n = 74) *M* [CI] (SD)	T2 (n = 59) *M* [CI] (SD)	T3 (*n* = 39) M [CI] (SD)	TI-T2 *t*_55_	*P*	*d*	T1-T3 *t*_35_	*P*	*d*
SDQ	Emotional symp.	4.4 [3.8-4.9] (2.3)	4.1 [3.5-4.8] (2.5)	3.9 [3.2-4.6] (2.1)	1.3	.19	0.18	1.2	0.22	0.21
	Conduct problems	2.6 [2.2-3.0] (1.7)	2.4 [2.0-2.7] (1.5)	1.8 [1.4-2.3] (1.3)	1.9	.06	0.26	1.0	0.31	0.16
	Hyperactivity	5.0 [4.4-5.5] (2.4)	4.3 [3.7-4.8] (2.0)	3.7 [3.1-4.3] (1.9)	3.7	<.01	0.49	1.9	0.07	0.31
	Peer relationship pr.	3.5 [3.0-4.0] (2.1)	3.3 [2.8-3.9] (2.2)	3.3 [2.7-4.0] (2.0)	0.2	.81	0.03	0.2	0.83	0.04
	Prosocial behavior	6.6 [6.1-7.2] (2.5)	7.0 [6.3-7.6] (2.4)	7.5 [6.7-8.2] (2.3)	−1.4	.18	0.18	−1.8	0.07	0.31
	Total	15.5 [14.3-16.7] (5.0)	14.1 [12.9-15.3] (4.7)	12.7 [11.3-14.1] (4.3)	3.0	<.01	0.41	3.1	0.07	0.31
	Impact factor	6.8 [5.9-7.6] (3.4)	4.4 [3.3-5.6] (4.3)	5.1 [3.9-6.3] (3.7)	3.7	<.01	0.54	1.9	0.07	0.36
		T1 (n = 66)	T2 (n = 50)	T3 (n = 36)	*t*_44_			*t*_32_		
HAD anxiety		7.4 [6.3-8.6] (4.7)	7.1 [5.8-8.5] (4.7)	6.1 [4.9-7.3] (3.3)	2.5	.02	0.37	2.5	0.02	0.43
Depression		6.7 [5.6-7.9] (4.5)	4.9 [4.0-5.9] (3.3)	5.1 [4.1-6.2] (3.1)	2.4	.02	0.36	2.4	0.02	0.42
		T1 (n = 68-71)	T2 (n = 57)	T3 (n = 39)	*i*_52_			*t*_34_		
LoL	One year ago	4.0 [3.4-4.5] (2.3)	3.6 [3.1-4.1] (2.0)	4.2 [3.6-4.8] (1.9)	1.6	.11	0.23	0.5	0.65	0.08
	Present time	4.9 [4.5-5.4] (1.9)	6.3 [5.8-6.8] (1.9)	6.2 [5.6-6.8] (1.8)	−4.6	<.01	0.63	−3.0	<0.01	0.51
	One year from now	6.5 [5.9-7.1] (2.5)	7.4 [6.8-7.9] (2.2)	7.4 [6.8-8.1] (2.0)	−2.8	<.01	0.39	−2.5	0.02	0.42

Mean, (95% confidence interval IKIJ around the mean, and standard deviation (SD) for self-reports of SDQ, HAD, and Ladder of Life (LoL) at pre (T1), post (T2) and follow up (T3).

#### SDQ-S

Youth total difficulties decreased from T1 to T2 and from T1 to T3. Among those who answered SDQ both at T1 and T2 and at T1 and T3, there was a decrease in impact over time. All these comparisons were statistically significant (*P* < .05) with small or medium effect sizes. However, the change in emotional symptoms was not as clear, with smaller effect sizes and non-statistically significant differences. The proportion of youths with a total difficulties score (SDQ Total) over the cut-off was 38% before treatment, 36% after treatment, and 26% at follow-up.

#### HADS

Youth anxiety and depression decreased over time both from T1 to T2 and from T1 to T3. Effect sizes were small (*d* = 0.27-0.42) but statistically significant. The proportion with a possible or probable anxiety disorder decreased over time. Possible cases with an anxiety disorder (≥8) were 42.4% before treatment, 40.0% after treatment, and 36.1% at follow-up. Probable cases (≥11) were 24.2% before treatment, 16.0% after treatment, and 11.1% at follow-up. Possible cases with a depression disorder were 39.4% before treatment, 18.0% after treatment, and 22.2% at follow-up. Cases with a probable depression disorder were 19.7% before treatment, 8.0% after treatment, and 5.8% at follow-up.

#### Ladder of life

Quality of life improved over time, both when T1 was compared to T2 and when T1 was compared to T3. Effect sizes were medium, and the differences were statistically significant.

### Parent Rating of Youth Mental Health

#### SDQ-F

Parent rating of youth mental health, rated with the SDQ, showed an improvement over time (Table 3). Both the Total Difficulties Score and Impact Factor decreased—the differences were statistically significant and effect sizes for Total Difficulties Score and Impact Factor were small to large: 0.48-0.85 (Cohen’s *d*). There were a total of 14 comparisons over time of parent rating of SDQ, including both subscales and total score. Of these 14 comparisons, 2 were classified as no effect, 7 as small, 4 as medium, and 1 as large. Among these ratings, a decrease in emotional symptoms over time can be seen both from mother and father rating with small to medium effect sizes (*d* = 0.35-0.51). Of the mothers’ ratings, 75% of the youths had a total difficulty score over cut-off before treatment, 56% after treatment, and 49% at follow-up. Of the fathers’ ratings, 62% of the youths had a total score over cut-off before treatment, 37% after treatment, and 46% at follow-up.

**Table 3.** Parent Rated-Parent 1 (Mother) and Parent 2 (Father).

			T1 (n = 79) *M* [CI] (SD)	T2 (n = 55) *M* [CI] (SD)	T3 (n = 37) *M* [CI] (SD)	TI-T2 *t*_52_	*P*	*d*	TI-T3 *t*_34_	*P*	*d*
Mother	SDQ	Emotional symp.	5.5 [5.0-6.0] (2.4)	4.5 [3.8-5.1] (2.4)	4.1 [3.2-4.9] (2.6)	3.2	<.01	0.44	2.4	.02	0.40
		Conduct problems	2.5 [2.1-2.9] (1.8)	2.0 [1.5-2.4] (1.7)	2.0 [1.4-2.6] (1.9)	3.0	<.01	0.42	2.5	.02	0.42
		Hyperactivity	4.8 [4.3-5.4] (2.5)	4.5 [4.0-5.1] (2.2)	3.8 [3.1-4.5] (2.2)	1.30	.20	0.18	2.2	.04	0.37
		Peer relationship pr.	3.8 [3.3-4.4] (2.4)	3.8 [3.1-4.4] (2.5)	3.2 [2.4-4.0] (2.4)	1.9	.06	0.27	2.1	.04	0.36
		Prosocial behavior	6.5 [6.0-7.1] (2.3)	6.5 [5.9-7.2] (2.4)	6.3 [5.4-7.1] (2.5)	−1.3	.20	0.19	−0.4	.68	0.07
		Total	16.7 [15.5-17.8] (5.1)	14.7 [13.4-16.1] (5.1)	13.0 [11.2-14.9] (5.6)	3.5	<.01	0.48	3.3	<.01	0.55
		Impact factor	10.5 [9.7-11.4] (3.8)	7.9 [6.7-9.2] (4.5)	8.9 [7.7-10.2] (3.8)	5.4	<.01	0.75	3.5	<.01	0.58
			T1 (n = 57)	T2 (n = 49)	T3 (n = 33)	*t*_41_			*t*_27_		
	HAD	Anxiety	9.0 [8.1-10.0] (3.9)	8.0 [6.7-9.3] (4.5)	8.1 [6.5-9.7] (4.5)	2.8	<.01	0.42	1.4	.16	0.27
		Depression	7.0 [5.8-8.2] (4.9)	6.3 [5.1-7.5] (4.2)	5.3 [3.9-6.8] (4.1)	2.4	.02	0.37	2.1	.05	0.39
			T1 (n = 54)	T2 (n = 49)	T3 (n = 34)	*t*_41_			*t*_29_		
	LoL	One year ago	4.4 [3.8-4.9] (2.2)	4.0 [3.4-4.5] (1.8)	4.7 [4.1-5.3] (1.6)	1.1	.29	0.16	0.6	.56	0.11
		Present time	5.1 [4.6-5.6] (2.1)	5.4 [4.9-6.0] (2.0)	6.4 [5.8-7.1] (1.8)	−2.2	.03	0.34	−3.6	<.01	0.66
		One year from now	7.0 [6.5-7.5] (2.0)	7.3 [6.7-7.8] (1.9)	7.6 [6.9-8.3] (2.0)	−1.7	.09	0.27	−1.6	.12	0.30
			T1 (n = 50) *M* [CI] (SD)	T2 (n = 35) *M* [CI] (SD)	T3 (n = 26) *M* [CI] (SD)	TI-T2 *t*_33_			TI-T3 *t*_24_		
Father	SDQ	Emotional symp.	4.7 [4.2-5.3] (2.2)	3.7 [2.9-4.6] (2.4)	3.5 [2.7-4.2] (1.9)	2.9	<.01	0.51	1.7	.10	0.35
		Conduct problems	2.6 [2.1-3.2] (2.1)	2.1 [1.3-2.7] (1.8)	1.8 [1.2-2.5] (1.5)	1.8	.09	0.30	2.0	.06	0.40
		Hyperactivity	4.7 [4.1-5.3] (2.4)	3.7 [3.1-4.4] (1.8)	3.7 [2.9-4.6] (2.0)	2.9	<.01	0.49	2.3	.03	0.46
		Peer relationship pr.	3.6 [3.0-4.1] (2.1)	3.7 [2.9-4.4] (2.3)	3.4 [2.5-4.2] (2.1)	0.1	.92	0.02	−0.2	.81	0.05
		Prosocial behavior	6.1 [5.5-6.7] (2.4)	6.7 [5.9-7.5] (2.3)	6.8 [5.9-7.8] (2.3)	−2.1	.04	0.36	−2.6	.02	0.52
		Total	15.6 [14.1-17.1] (5.7)	13.2 [11.6-14.9] (4.8)	12.4 [10.7-14.1] (4.2)	3.0	<.01	0.51	2.4	.02	0.48
		Impact factor	10.0 [9.0-11.0] (3.9)	7.8 [6.2-9.4] (4.7)	7.5 [5.8-9.2] (4.1)	4.6	<.01	0.78	4.2	<.01	0.85
			T1 (n = 51)	T2 (n = 34)	T3 (n = 23)	*t*_31_			*t*_22_		
	HAD	Anxiety	7.0 [5.7-8.3] (4.5)	6.5 [5.0-8.1] (4.5)	6.2 [4.7-7.8] (3.6)	1.3	.20	0.23	1.3	.20	0.28
		Depression	6.2 [5.1-7.4] (4.0)	5.2 [3.8-6.6] (4.1)	5.8 [4.1-7.6] (4.0)	1.3	.19	0.24	0.0	1.0	0.0
			T1 (n = 48-50)	T2 (n = 36)	T3 (n = 24)	*t*_33_			*t*_22_		
	LoL	One year ago	5.1 [4.6-5.7] (1.8)	4.9 [4.1-5.7] (2.3)	5.4 [4.7-6.0] (1.6)	0.7	.47	0.13	−1.6	.13	0.34
		Present time	5.7 [5.2-6.2] (1.7)	5.9 [5.3-6.5] (1.7)	6.3 [5.4-7.1] (2.0)	−0.4	.72	0.06	−2.0	.06	0.41
		One year from now	7.1 [6.6-7.6] (1.8)	7.1 [6.5-7.7] (1.8)	7.3 [6.6-8.1] (1.7)	−0.2	.84	0.04	−1.7	.10	0.36

Mean (M), 95% confidence interval [CI] around the mean, and standard deviation (SD) for parent rating of SDQ, HAD, and Ladder of Life (LoL) at pre (TI), post (T2) and follow up (T3).

### Parent Rating of Their Own Mental Health

Before treatment, mental health in the form of anxiety, depression, and life satisfaction was worse for mothers than fathers, a difference that was not statistically significant. Mental health also changed over time for mothers more than for fathers.

Mothers’ mental health improved over time: degree of anxiety and depression decreased (Table 3) and quality of life increased. Differences in anxiety, depression, and quality of life (Ladder of life—present time) were all statistically significant, except anxiety from T1 to T3 (*P* = .16), and the effect sizes were small to medium (*d* = 0.27-0.66). The proportion with a probable anxiety disorder^27^ decreased from 34.3% before treatment to 32.7% after treatment and 30.3% at follow-up. The proportion with a probable depression disorder decreased from 26.9% before treatment to 16.3% after treatment and 9.1% at follow-up.

Fathers’ mental health improved over time, but most of the comparisons were not statistically significant (except Ladder of Life—Present time from T1 to T3) and effect sizes were non-existing to small (*d* = 0.0-0.41). Effect sizes for anxiety were small. The difference in depression was small from T1 to T2 and non-existing from T1 to T3. The difference in quality of life was non-existing from T1 to T2 and small from T1 to T3. The proportion with a probable anxiety disorder was 27.5% before treatment, 20.6% after treatment, and 13.0% at follow-up. The proportion with a probable depression disorder was 15.7% before treatment, 8.8% after treatment, and 13.0% at follow-up.

## Discussion

This study evaluates the outcome of a CBT-based treatment intervention for SR—that is, HSP. This study is part of MDI’s routine follow-up, which is intended to help caregivers control the quality of their treatment centers through self-monitoring.^19^ Outcome was assessed by measuring school attendance, youth mental health, and parent mental health. After treatment and at follow-up, school attendance increased, mental health among of the youth and mothers improved, and mental health of fathers slightly improved.

Participants went to school 4 times as much after treatment and 7 times as much at follow-up than before treatment. Before treatment, a majority of youths were totally absent from school and had been absent for a long time, longer than in other studies of school refusal (eg, Heyne et al^37^ and King et al^38^): 76% of the youths were totally absent from school before treatment, 41% after treatment, and 27% at follow-up. That is, for many of these youths, the period of total absence was interrupted. Improved mental health was evident in youth self-reports, with the largest change in a better quality of life. In research of SR, anxiety is a commonly used primary outcome measure.^24^ In this study, the proportion of youth with a possible or probable anxiety disorder decreased. The improvement in mental health among youths is even more distinguishable in the parent ratings, with larger effect sizes. Furthermore, for parent mental health, a change can also be seen: decreased anxiety and depression and improved quality of life. Fathers rated their mental health as better than the mothers before treatment, and their mental health did not change as much as the mothers’ mental health after treatment. After discussions with school staff, these students often were given individualized schedules that reduced the number of lessons at post-treatment and follow-up.

This result confirms the changes in mental health reported in Strömbeck et al,^39^ with the addition of a third measuring point—follow-up. Furthermore, here the focus is on the mental health of the youths as well as their parents. Previous studies have found increased school attendance after treatment, but no change in anxiety,^14^ whereas this study found a decrease in anxiety levels. Measures of mental health in other intervention studies have generated effect sizes from non-existing (*d* = −0.07) to large (*d* = 4.63) when different treatment methods are compared.^40^

According to Goodman and Scott,^41^ about 70% of SR interventions are a ‘success’; however, they do not clearly define what they mean by success. The best chance of a successful outcome is with younger children, when the symptoms are less severe, and when it is possible to intervene at an earlier stage. Better outcomes have been reported for younger students (7-11 years old) compared to older students (12-18 year). These findings might be related to the fact that absence from school is often greater for the older group and the degree of depression could also be higher for the older group.^42^ In this study of HSP, most of the participants were 12 years old or older, the absence had lasted for a long time, and many had clinically significant levels of symptoms (over cut-offs); in other words, this group is of risk of a less fortunate outcome.

Effect sizes could be compared to earlier studies. Pina et al^40^ report school attendance rates of 30% before treatment and 75% after treatment, whereas our study reports school attendance of 4% before treatment, 17% after treatment, and 28% at follow-up. There is a large difference prior to treatment (30% vs 4%). Although the actual increase is larger in the former (45% compared to 13.1% for HSP), the percentage change is larger in the latter. Moreover, attendance was doubled in Pina et al^40^ and quadrupled in this study.

The population seems to be different from those reported in previous studies. For example, King et al^38^ studied a sample that could be classified as SR according to Berg’s^32^ definition. In addition, school attendance was measured during the previous 3 months before intervention. In this period, 10 participants were totally absent, 17 were partially/intermittently absent, and 7 were actually in school but with a high degree of anxiety. At the initiation of HSP, only a small proportion of students (19%) had not been absent more than half of the lessons during the year before the intervention, a condition that causes the primary problem (ie, absence from school) to create secondary problems (eg, lower levels of mental health, social isolation, social phobia, and difficulties leaving home).^4^ Therefore, this group is also in need of a more complex intervention where the return to school has to be gradual.^17,41^ The longer the absence, the harder it is for youth to return to school.^17^ That is, it is not surprising that the proportion of students reaching an acceptable of school attendance is not higher.

### Strengths and Weaknesses with this Study

This study, the first outcome study of HSP, was conducted in a natural environment without the influence of researchers other than the standard evaluation procedure within MDI. The strength of this design is that the intervention is evaluated in real practice (effectiveness), without the control characteristic of efficacy studies. Furthermore, in comparison with similar studies (eg, King et al^43^ and McShane et al^44^), the sample size is also relatively large. Weaknesses of the study include a lack of a control group, randomization, and measures of treatment fidelity. Because of this weakness in the research design, it is not possible to know the impact of HSP and what caused the change. Risk of bias because of attrition also causes insecurity regarding representativity of the sample (sampling bias), which should be prevented as much as possible. Within MDI, efforts are being made to reduce attrition rates (eg, education and assistance from social workers who are responsible for data collection at each treatment unit).

School attendance was measured in 2 ways. The first assesses the number of lessons in the schedule and how many of these students attended school during the previous 3 weeks. The second estimates the number of lessons in the schedule during the previous 6 months and what proportion of these lessons the student attended. This estimation could be done by school staff, a parent, or a digital system for documentation where school attendance is registered. These 2 ways of measuring attendance have their advantages and disadvantages. The advantage with the latter is that the data collection becomes easier and so the amount of missing data is decreased, but the disadvantage is that the variable becomes less sensitive to change because of the long-time interval. As the 2 measuring methods gave the same result, the methods could be assessed as equivalent and a proof of concurrent validity.

### Future Studies

This first outcome study of HSP should be followed by well-controlled studies. More studies are needed despite the additional support for CBT-based interventions for SR and the initial support for HSP. In a systematic overview and meta-analysis, Maynard et al^14,18^ concluded that more well-designed studies with larger sample sizes and longer follow-ups are needed. Because of the heterogeneity of the population of SR^14,45^ and the different combinations of risk and protective factors, future studies should compare how youths at HSP differ from samples in earlier studies of SR interventions.

The importance of using a common set of methods for assessment^46^ and outcome^24^ has previously been emphasized, a study design feature that could contribute to the assessment of the role of absence and the role of the parent and the school in the treatment planning.^47^ These instruments already exist, including the School Refusal Assessment Scale (SRAS).^48^ New instruments are being developed such as the Inventory of School Attendance Problems (ISAP; Knollman et al^49^), and SChool REfusal EvaluatioN Scale (SCREEN).^50^ These instruments can help the clinician and researchers identify and classify school attendance problems. A thorough description of the target group and a standardized way of evaluating outcome should help facilitate comparisons between methods, contexts, and subgroups.^24^

This study seems to measure the right constructs as the treatment goals for HSP and other interventions are to increase school attendance and to decrease anxiety. Moreover, the study uses the appropriate measuring points—pre-test, post-test, and follow-up—to determine whether the effect is long lasting. However, standardizing how to measure outcomes of interventions needs further investigations,^51,52^ such as Heyne et al’s^24^ review of the way SR interventions have been evaluated over the past 40 years.

## Conclusion

School absenteeism can be classified as either problematic or unproblematic. If sufficient help is not received, acute absence with a recent onset can turn into chronic absence and ultimately drop out.^46^ The longer a student is absent, the greater the need for comprehensive interventions. For the young people at HSP, their absence was long term so the return to school had to be gradual^17,41^ and accompanied with intense support.^4^

According to the Education Act, the different needs of students should be considered. HSP’s protocols include cooperating with the schools to create a reduced course of studies for youth attempting to re-integrate into the school environment.^3^ A successful intervention also includes adjustments at school. Without adjustments at school, the intervention’s full potential will not be reached as the students will return to the same environment that may have led to their SR. Many of the young persons at HSP enter specialized units that combine treatment and school. Others attend resource schools or continue with adjusted education, strategies that could point to the fact that the old school did not adjust the environment to meet the needs of the returning students.
